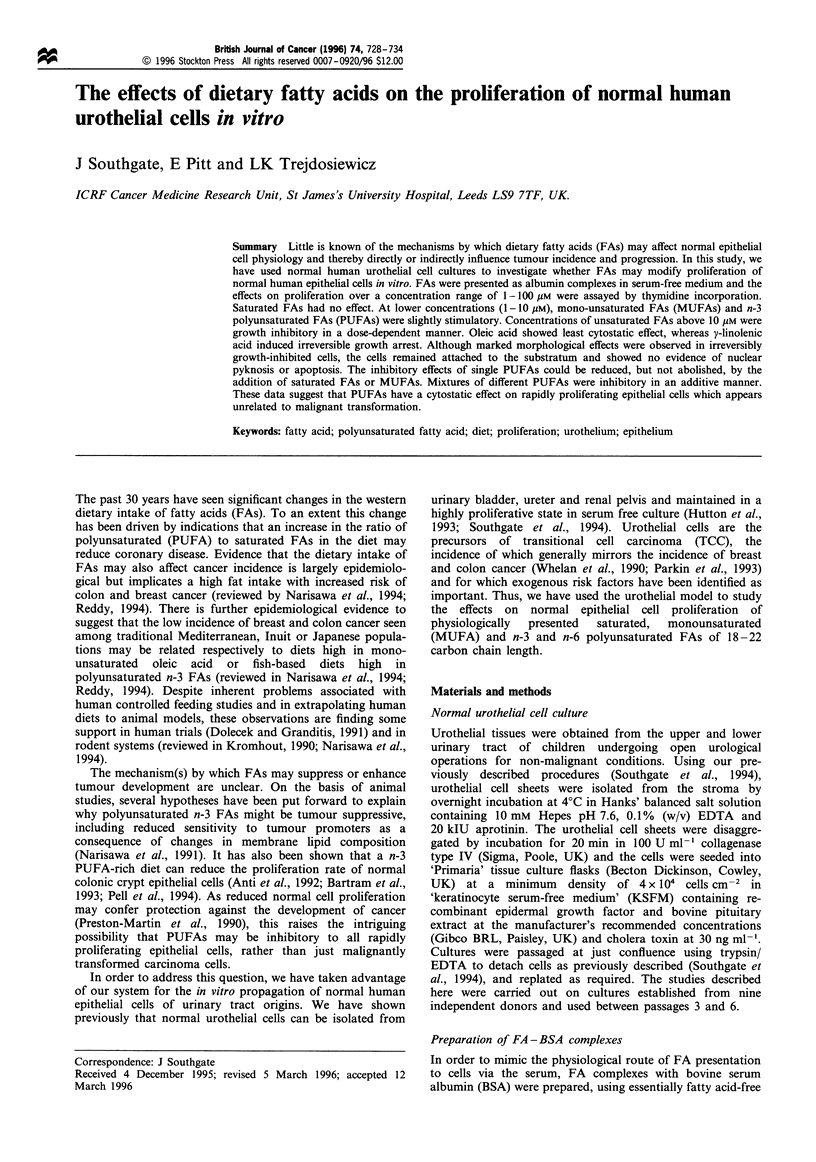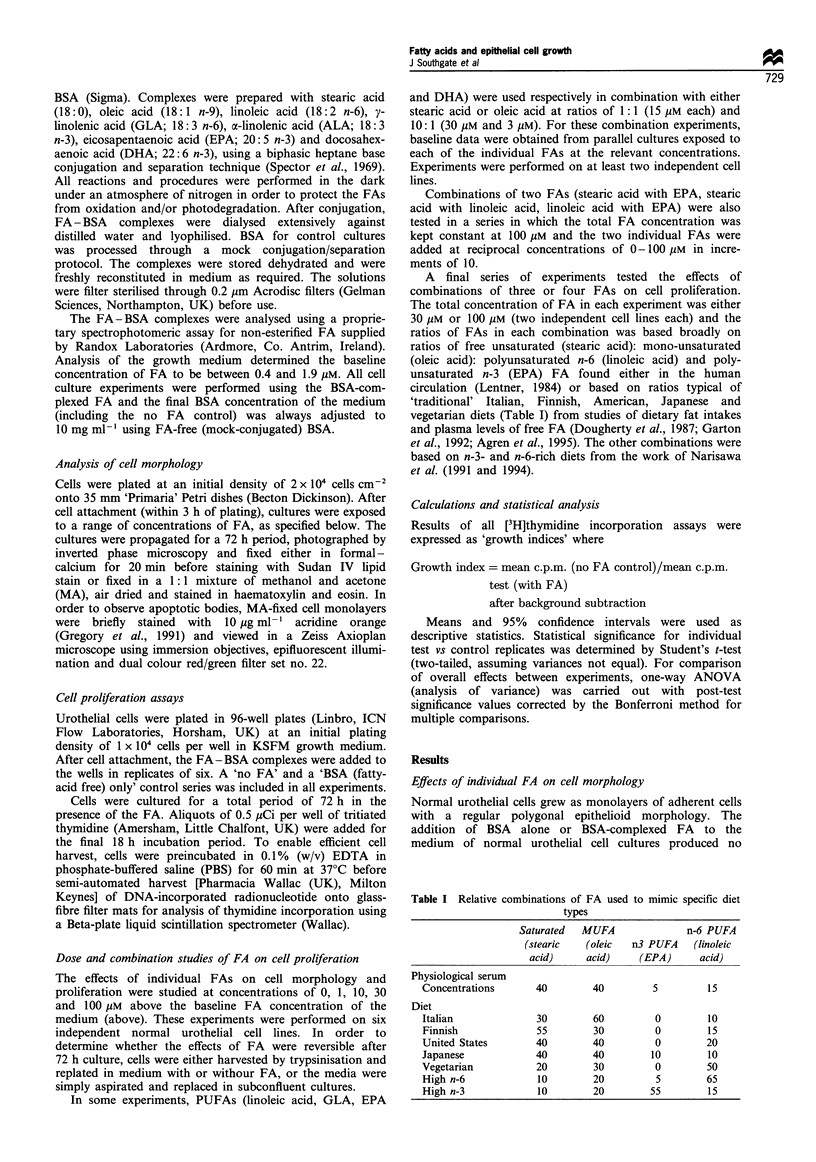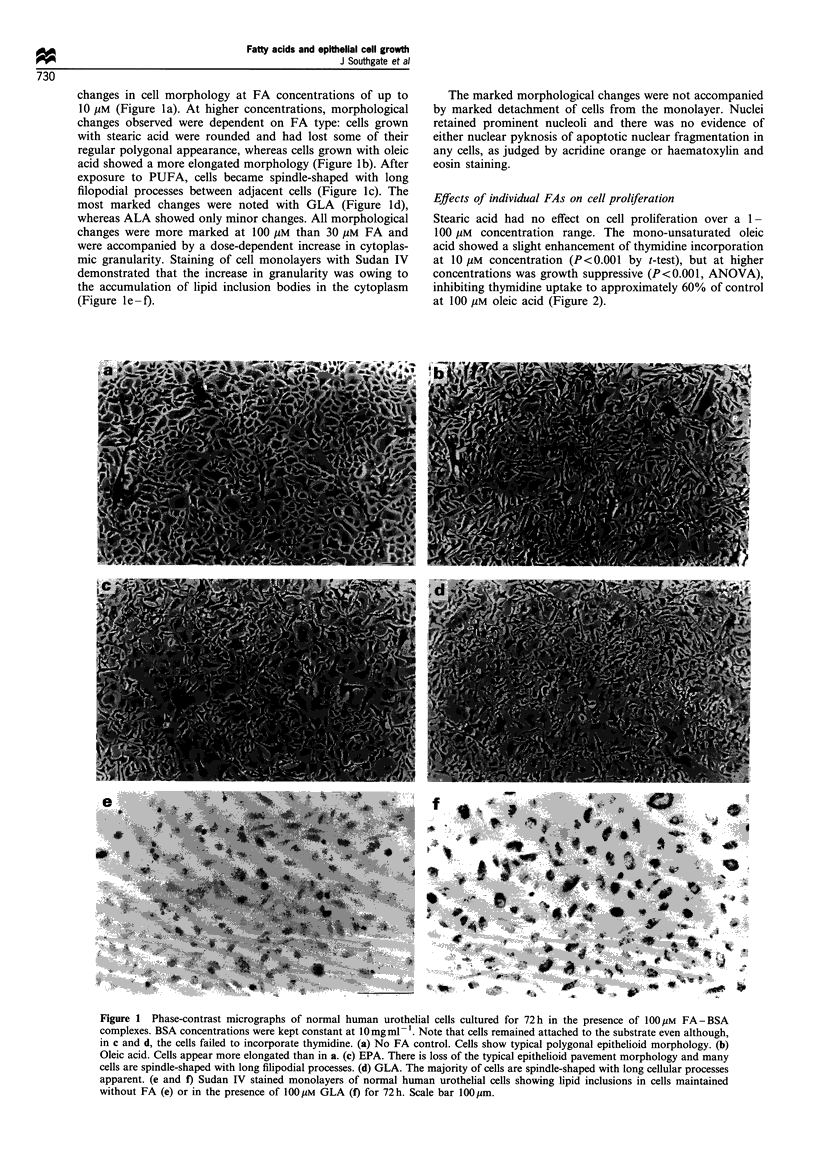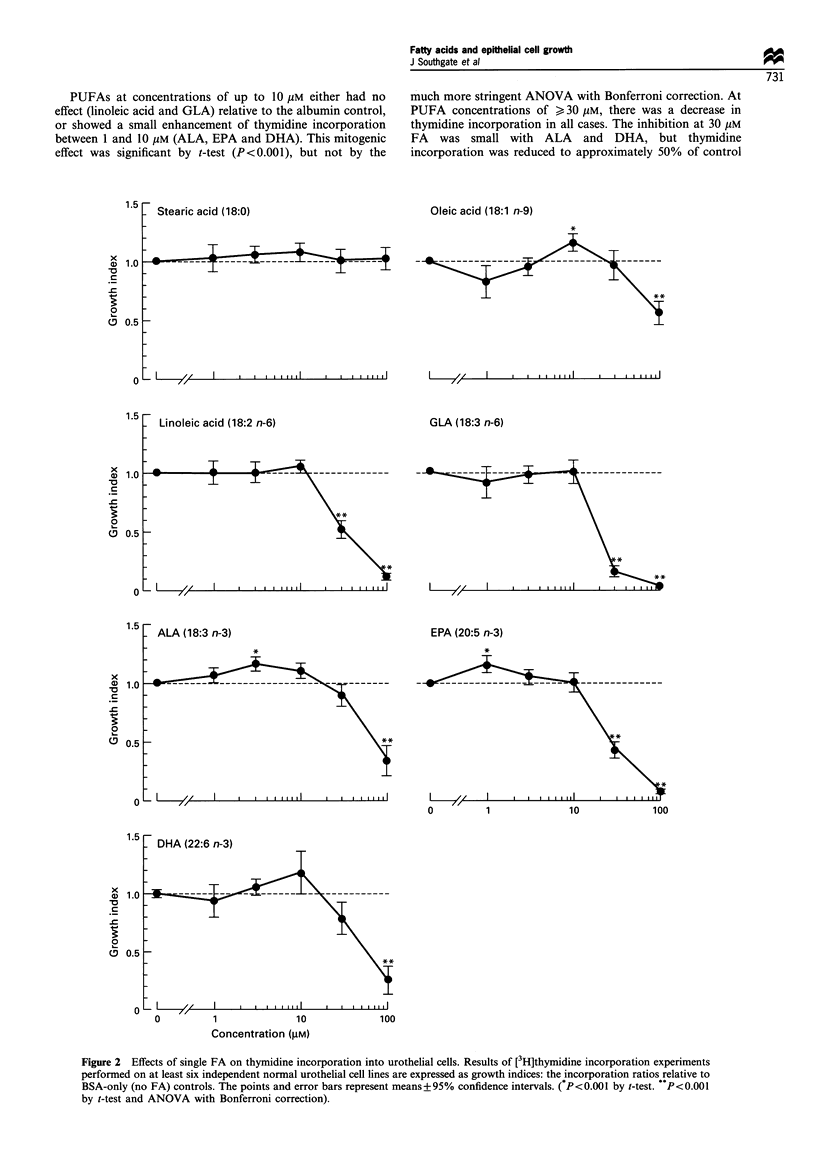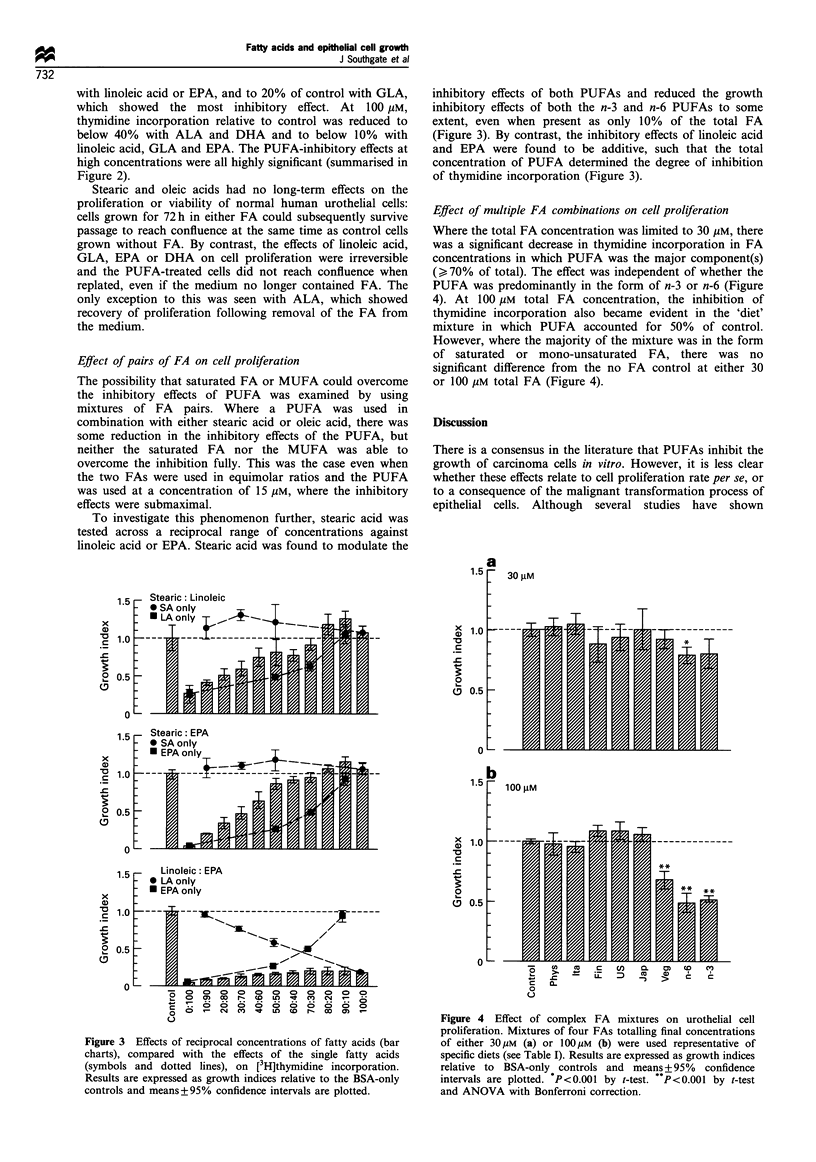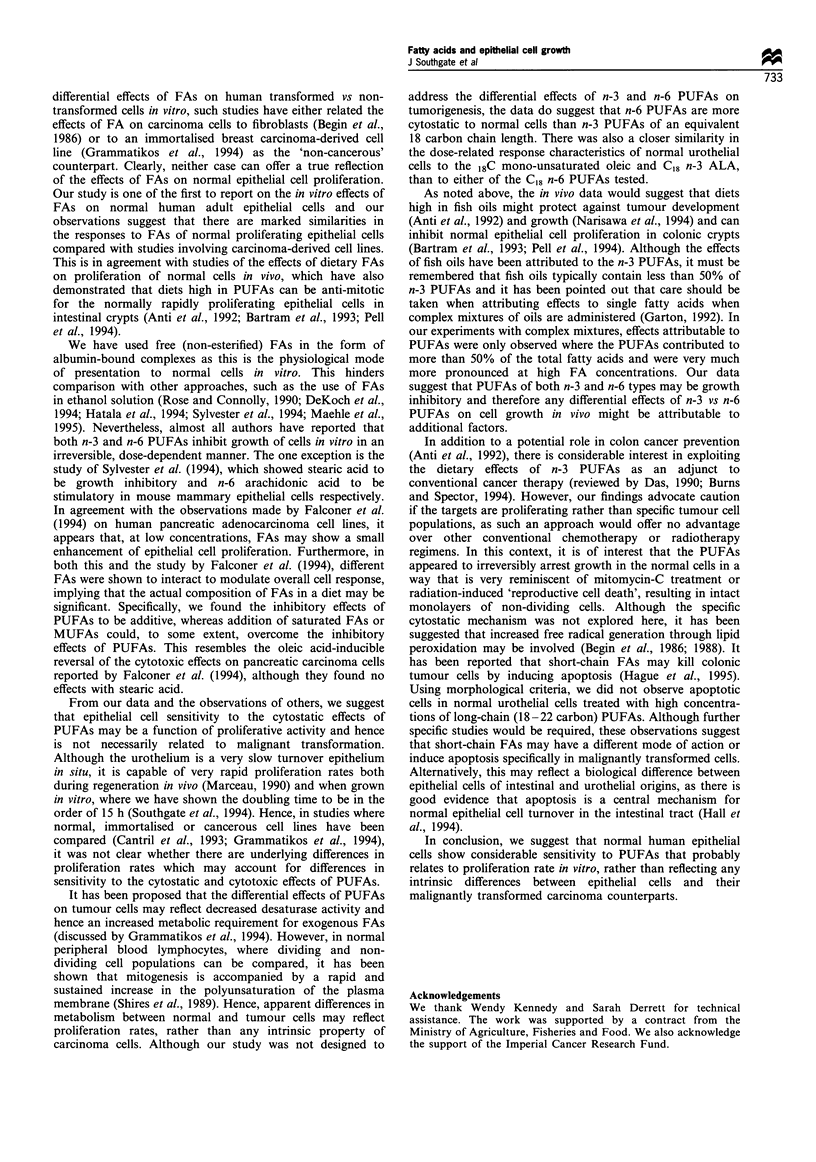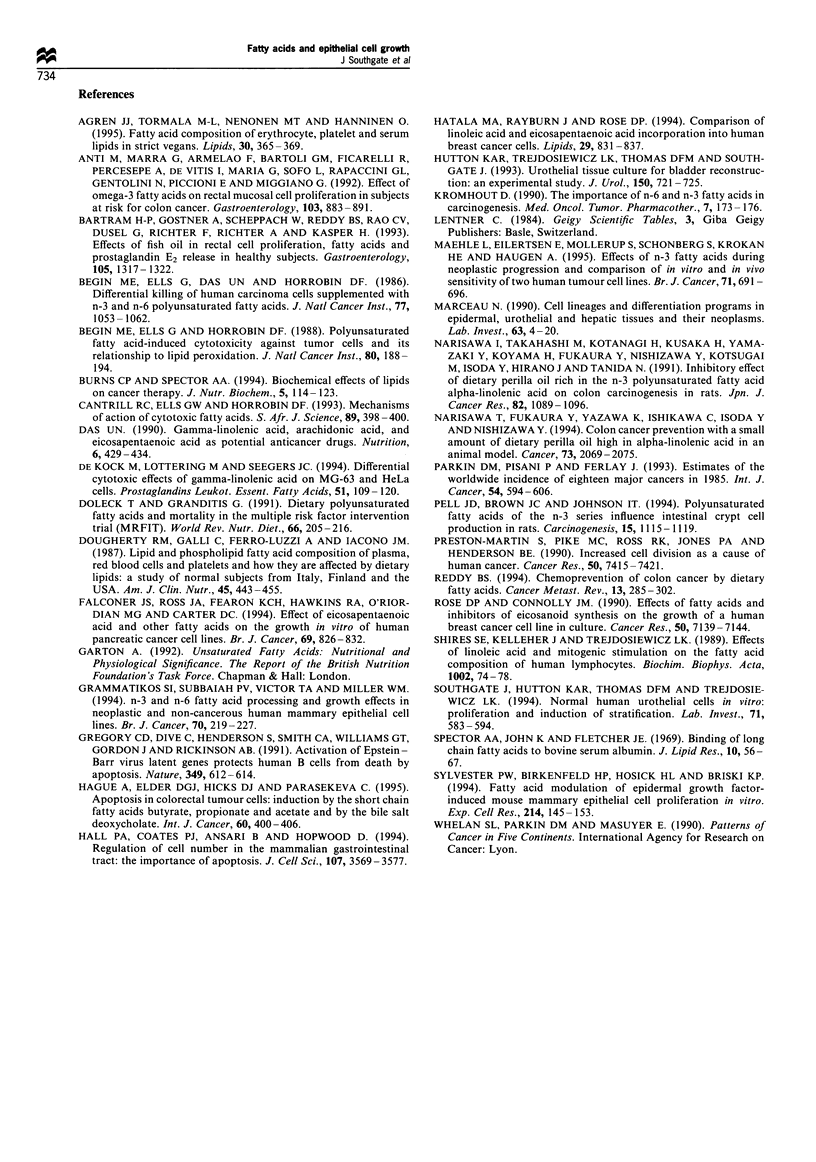# The effects of dietary fatty acids on the proliferation of normal human urothelial cells in vitro.

**DOI:** 10.1038/bjc.1996.429

**Published:** 1996-09

**Authors:** J. Southgate, E. Pitt, L. K. Trejdosiewicz

**Affiliations:** ICRF Cancer Medicine Research Unit, St James's University Hospital, Leeds, UK.

## Abstract

**Images:**


British Journal of Cancer (1996) 74, 728-734
© 1996 Stockton Press All rights reserved 0007-0920/96 $12.00

### The effects of dietary fatty acids on the proliferation of normal human urothelial cells *in vitro*

J Southgate, E Pitt and LK Trejdosiewicz

*ICRF Cancer Medicine Research Unit, St James's University Hospital, Leeds LS9 7TF, UK.*

**Summary** Little is known of the mechanisms by which dietary fatty acids (FAs) may affect normal epithelial cell physiology and thereby directly or indirectly influence tumour incidence and progression. In this study, we have used normal human urothelial cell cultures to investigate whether FAs may modify proliferation of normal human epithelial cells *in vitro*. FAs were presented as albumin complexes in serum-free medium and the effects on proliferation over a concentration range of 1-100 $\mu$M were assayed by thymidine incorporation. Saturated FAs had no effect. At lower concentrations (1-10 $\mu$M), mono-unsaturated FAs (MUFAs) and *n*-3 polyunsaturated FAs (PUFAs) were slightly stimulatory. Concentrations of unsaturated FAs above 10 $\mu$M were growth inhibitory in a dose-dependent manner. Oleic acid showed least cytostatic effect, whereas $\gamma$-linolenic acid induced irreversible growth arrest. Although marked morphological effects were observed in irreversibly growth-inhibited cells, the cells remained attached to the substratum and showed no evidence of nuclear pyknosis or apoptosis. The inhibitory effects of single PUFAs could be reduced, but not abolished, by the addition of saturated FAs or MUFAs. Mixtures of different PUFAs were inhibitory in an additive manner. These data suggest that PUFAs have a cytostatic effect on rapidly proliferating epithelial cells which appears unrelated to malignant transformation.

**Keywords:** fatty acid; polyunsaturated fatty acid; diet; proliferation; urothelium; epithelium

The past 30 years have seen significant changes in the western dietary intake of fatty acids (FAs). To an extent this change has been driven by indications that an increase in the ratio of polyunsaturated (PUFA) to saturated FAs in the diet may reduce coronary disease. Evidence that the dietary intake of FAs may also affect cancer incidence is largely epidemiological but implicates a high fat intake with increased risk of colon and breast cancer (reviewed by Narisawa *et al.*, 1994; Reddy, 1994). There is further epidemiological evidence to suggest that the low incidence of breast and colon cancer seen among traditional Mediterranean, Inuit or Japanese populations may be related respectively to diets high in monounsaturated oleic acid or fish-based diets high in polyunsaturated *n*-3 FAs (reviewed in Narisawa *et al.*, 1994; Reddy, 1994). Despite inherent problems associated with human controlled feeding studies and in extrapolating human diets to animal models, these observations are finding some support in human trials (Dolecek and Granditis, 1991) and in rodent systems (reviewed in Kromhout, 1990; Narisawa *et al.*, 1994).

The mechanism(s) by which FAs may suppress or enhance tumour development are unclear. On the basis of animal studies, several hypotheses have been put forward to explain why polyunsaturated *n*-3 FAs might be tumour suppressive, including reduced sensitivity to tumour promoters as a consequence of changes in membrane lipid composition (Narisawa *et al.*, 1991). It has also been shown that a *n*-3 PUFA-rich diet can reduce the proliferation rate of normal colonic crypt epithelial cells (Anti *et al.*, 1992; Bartram *et al.*, 1993; Pell *et al.*, 1994). As reduced normal cell proliferation may confer protection against the development of cancer (Preston-Martin *et al.*, 1990), this raises the intriguing possibility that PUFAs may be inhibitory to all rapidly proliferating epithelial cells, rather than just malignantly transformed carcinoma cells.

In order to address this question, we have taken advantage of our system for the *in vitro* propagation of normal human epithelial cells of urinary tract origins. We have shown previously that normal urothelial cells can be isolated from urinary bladder, ureter and renal pelvis and maintained in a highly proliferative state in serum free culture (Hutton *et al.*, 1993; Southgate *et al.*, 1994). Urothelial cells are the precursors of transitional cell carcinoma (TCC), the incidence of which generally mirrors the incidence of breast and colon cancer (Whelan *et al.*, 1990; Parkin *et al.*, 1993) and for which exogenous risk factors have been identified as important. Thus, we have used the urothelial model to study the effects on normal epithelial cell proliferation of physiologically presented saturated, monounsaturated (MUFA) and *n*-3 and *n*-6 polyunsaturated FAs of 18-22 carbon chain length.

### Materials and methods

### *Normal urothelial cell culture*

Urothelial tissues were obtained from the upper and lower urinary tract of children undergoing open urological operations for non-malignant conditions. Using our previously described procedures (Southgate *et al.*, 1994), urothelial cell sheets were isolated from the stroma by overnight incubation at 4°C in Hanks' balanced salt solution containing 10 mM Hepes pH 7.6, 0.1% (w/v) EDTA and 20 kIU aprotinin. The urothelial cell sheets were disaggregated by incubation for 20 min in 100 U ml$^{-1}$ collagenase type IV (Sigma, Poole, UK) and the cells were seeded into 'Primaria' tissue culture flasks (Becton Dickinson, Cowley, UK) at a minimum density of $4 \times 10^4$ cells cm$^{-2}$ in 'keratinocyte serum-free medium' (KSFM) containing recombinant epidermal growth factor and bovine pituitary extract at the manufacturer's recommended concentrations (Gibco BRL, Paisley, UK) and cholera toxin at 30 ng ml$^{-1}$. Cultures were passaged at just confluence using trypsin/EDTA to detach cells as previously described (Southgate *et al.*, 1994), and replated as required. The studies described here were carried out on cultures established from nine independent donors and used between passages 3 and 6.

### *Preparation of FA-BSA complexes*

In order to mimic the physiological route of FA presentation to cells via the serum, FA complexes with bovine serum albumin (BSA) were prepared, using essentially fatty acid-free

Correspondence: J Southgate
Received 4 December 1995; revised 5 March 1996; accepted 12 March 1996

BSA (Sigma). Complexes were prepared with stearic acid (18:0), oleic acid (18:1 *n*-9), linoleic acid (18:2 *n*-6), γ-linolenic acid (GLA; 18:3 *n*-6), α-linolenic acid (ALA; 18:3 *n*-3), eicosapentaenoic acid (EPA; 20:5 *n*-3) and docosahexaenoic acid (DHA; 22:6 *n*-3), using a biphasic heptane base conjugation and separation technique (Spector *et al.*, 1969). All reactions and procedures were performed in the dark under an atmosphere of nitrogen in order to protect the FAs from oxidation and/or photodegradation. After conjugation, FA–BSA complexes were dialysed extensively against distilled water and lyophilised. BSA for control cultures was processed through a mock conjugation/separation protocol. The complexes were stored dehydrated and were freshly reconstituted in medium as required. The solutions were filter sterilised through 0.2 μm Acrodisc filters (Gelman Sciences, Northampton, UK) before use.

The FA–BSA complexes were analysed using a proprietary spectrophotomeric assay for non-esterified FA supplied by Randox Laboratories (Ardmore, Co. Antrim, Ireland). Analysis of the growth medium determined the baseline concentration of FA to be between 0.4 and 1.9 μM. All cell culture experiments were performed using the BSA-complexed FA and the final BSA concentration of the medium (including the no FA control) was always adjusted to 10 mg $ml^{-1}$ using FA-free (mock-conjugated) BSA.

### *Analysis of cell morphology*

Cells were plated at an initial density of $2 \times 10^4$ cells $cm^{-2}$ onto 35 mm 'Primaria' Petri dishes (Becton Dickinson). After cell attachment (within 3 h of plating), cultures were exposed to a range of concentrations of FA, as specified below. The cultures were propagated for a 72 h period, photographed by inverted phase microscopy and fixed either in formal–calcium for 20 min before staining with Sudan IV lipid stain or fixed in a 1:1 mixture of methanol and acetone (MA), air dried and stained in haematoxylin and eosin. In order to observe apoptotic bodies, MA-fixed cell monolayers were briefly stained with 10 μg $ml^{-1}$ acridine orange (Gregory *et al.*, 1991) and viewed in a Zeiss Axioplan microscope using immersion objectives, epifluorescent illumination and dual colour red/green filter set no. 22.

### *Cell proliferation assays*

Urothelial cells were plated in 96-well plates (Linbro, ICN Flow Laboratories, Horsham, UK) at an initial plating density of $1 \times 10^4$ cells per well in KSFM growth medium. After cell attachment, the FA–BSA complexes were added to the wells in replicates of six. A 'no FA' and a 'BSA (fatty-acid free) only' control series was included in all experiments.

Cells were cultured for a total period of 72 h in the presence of the FA. Aliquots of 0.5 μCi per well of tritiated thymidine (Amersham, Little Chalfont, UK) were added for the final 18 h incubation period. To enable efficient cell harvest, cells were preincubated in 0.1% (w/v) EDTA in phosphate-buffered saline (PBS) for 60 min at 37°C before semi-automated harvest [Pharmacia Wallac (UK), Milton Keynes] of DNA-incorporated radionucleotide onto glass-fibre filter mats for analysis of thymidine incorporation using a Beta-plate liquid scintillation spectrometer (Wallac).

### *Dose and combination studies of FA on cell proliferation*

The effects of individual FAs on cell morphology and proliferation were studied at concentrations of 0, 1, 10, 30 and 100 μM above the baseline FA concentration of the medium (above). These experiments were performed on six independent normal urothelial cell lines. In order to determine whether the effects of FA were reversible after 72 h culture, cells were either harvested by trypsinisation and replated in medium with or withour FA, or the media were simply aspirated and replaced in subconfluent cultures.

In some experiments, PUFAs (linoleic acid, GLA, EPA and DHA) were used respectively in combination with either stearic acid or oleic acid at ratios of 1:1 (15 μM each) and 10:1 (30 μM and 3 μM). For these combination experiments, baseline data were obtained from parallel cultures exposed to each of the individual FAs at the relevant concentrations. Experiments were performed on at least two independent cell lines.

Combinations of two FAs (stearic acid with EPA, stearic acid with linoleic acid, linoleic acid with EPA) were also tested in a series in which the total FA concentration was kept constant at 100 μM and the two individual FAs were added at reciprocal concentrations of 0–100 μM in increments of 10.

A final series of experiments tested the effects of combinations of three or four FAs on cell proliferation. The total concentration of FA in each experiment was either 30 μM or 100 μM (two independent cell lines each) and the ratios of FAs in each combination was based broadly on ratios of free unsaturated (stearic acid): mono-unsaturated (oleic acid): polyunsaturated *n*-6 (linoleic acid) and polyunsaturated *n*-3 (EPA) FA found either in the human circulation (Lentner, 1984) or based on ratios typical of 'traditional' Italian, Finnish, American, Japanese and vegetarian diets (Table I) from studies of dietary fat intakes and plasma levels of free FA (Dougherty *et al.*, 1987; Garton *et al.*, 1992; Agren *et al.*, 1995). The other combinations were based on *n*-3- and *n*-6-rich diets from the work of Narisawa *et al.* (1991 and 1994).

### *Calculations and statistical analysis*

Results of all [$^3$H]thymidine incorporation assays were expressed as 'growth indices' where

Growth index = mean c.p.m. (no FA control)/mean c.p.m. test (with FA) after background subtraction

Means and 95% confidence intervals were used as descriptive statistics. Statistical significance for individual test *vs* control replicates was determined by Student's *t*-test (two-tailed, assuming variances not equal). For comparison of overall effects between experiments, one-way ANOVA (analysis of variance) was carried out with post-test significance values corrected by the Bonferroni method for multiple comparisons.

### Results

### *Effects of individual FA on cell morphology*

Normal urothelial cells grew as monolayers of adherent cells with a regular polygonal epithelioid morphology. The addition of BSA alone or BSA-complexed FA to the medium of normal urothelial cell cultures produced no

**Table I** Relative combinations of FA used to mimic specific diet types

| | *Saturated (stearic acid)* | *MUFA (oleic acid)* | n3 *PUFA (EPA)* | n-6 *PUFA (linoleic acid)* |
|---|---|---|---|---|
| Physiological serum Concentrations | 40 | 40 | 5 | 15 |
| Diet | | | | |
| Italian | 30 | 60 | 0 | 10 |
| Finnish | 55 | 30 | 0 | 15 |
| United States | 40 | 40 | 0 | 20 |
| Japanese | 40 | 40 | 10 | 10 |
| Vegetarian | 20 | 30 | 0 | 50 |
| High *n*-6 | 10 | 20 | 5 | 65 |
| High *n*-3 | 10 | 20 | 55 | 15 |

changes in cell morphology at FA concentrations of up to 10 μM (Figure 1a). At higher concentrations, morphological changes observed were dependent on FA type: cells grown with stearic acid were rounded and had lost some of their regular polygonal appearance, whereas cells grown with oleic acid showed a more elongated morphology (Figure 1b). After exposure to PUFA, cells became spindle-shaped with long filopodial processes between adjacent cells (Figure 1c). The most marked changes were noted with GLA (Figure 1d), whereas ALA showed only minor changes. All morphological changes were more marked at 100 μM than 30 μM FA and were accompanied by a dose-dependent increase in cytoplasmic granularity. Staining of cell monolayers with Sudan IV demonstrated that the increase in granularity was owing to the accumulation of lipid inclusion bodies in the cytoplasm (Figure 1e–f).

The marked morphological changes were not accompanied by marked detachment of cells from the monolayer. Nuclei retained prominent nucleoli and there was no evidence of either nuclear pyknosis of apoptotic nuclear fragmentation in any cells, as judged by acridine orange or haematoxylin and eosin staining.

### *Effects of individual FAs on cell proliferation*

Stearic acid had no effect on cell proliferation over a 1–100 μM concentration range. The mono-unsaturated oleic acid showed a slight enhancement of thymidine incorporation at 10 μM concentration ($P<0.001$ by *t*-test), but at higher concentrations was growth suppressive ($P<0.001$, ANOVA), inhibiting thymidine uptake to approximately 60% of control at 100 μM oleic acid (Figure 2).

**Figure 1** Phase-contrast micrographs of normal human urothelial cells cultured for 72 h in the presence of 100 μM FA–BSA complexes. BSA concentrations were kept constant at 10 mg ml$^{-1}$. Note that cells remained attached to the substrate even although, in **c** and **d**, the cells failed to incorporate thymidine. (**a**) No FA control. Cells show typical polygonal epithelioid morphology. (**b**) Oleic acid. Cells appear more elongated than in **a**. (**c**) EPA. There is loss of the typical epithelioid pavement morphology and many cells are spindle-shaped with long filipodial processes. (**d**) GLA. The majority of cells are spindle-shaped with long cellular processes apparent. (**e** and **f**) Sudan IV stained monolayers of normal human urothelial cells showing lipid inclusions in cells maintained without FA (**e**) or in the presence of 100 μM GLA (**f**) for 72 h. Scale bar 100 μm.

PUFAs at concentrations of up to 10 μM either had no effect (linoleic acid and GLA) relative to the albumin control, or showed a small enhancement of thymidine incorporation between 1 and 10 μM (ALA, EPA and DHA). This mitogenic effect was significant by t-test ($P<0.001$), but not by the much more stringent ANOVA with Bonferroni correction. At PUFA concentrations of ⩾30 μM, there was a decrease in thymidine incorporation in all cases. The inhibition at 30 μM FA was small with ALA and DHA, but thymidine incorporation was reduced to approximately 50% of control

**Figure 2** Effects of single FA on thymidine incorporation into urothelial cells. Results of [$^3$H]thymidine incorporation experiments performed on at least six independent normal urothelial cell lines are expressed as growth indices: the incorporation ratios relative to BSA-only (no FA) controls. The points and error bars represent means±95% confidence intervals. ($^*P<0.001$ by t-test. $^{**}P<0.001$ by t-test and ANOVA with Bonferroni correction).

with linoleic acid or EPA, and to 20% of control with GLA, which showed the most inhibitory effect. At 100 $\mu$M, thymidine incorporation relative to control was reduced to below 40% with ALA and DHA and to below 10% with linoleic acid, GLA and EPA. The PUFA-inhibitory effects at high concentrations were all highly significant (summarised in Figure 2).

Stearic and oleic acids had no long-term effects on the proliferation or viability of normal human urothelial cells: cells grown for 72 h in either FA could subsequently survive passage to reach confluence at the same time as control cells grown without FA. By contrast, the effects of linoleic acid, GLA, EPA or DHA on cell proliferation were irreversible and the PUFA-treated cells did not reach confluence when replated, even if the medium no longer contained FA. The only exception to this was seen with ALA, which showed recovery of proliferation following removal of the FA from the medium.

### *Effect of pairs of FA on cell proliferation*

The possibility that saturated FA or MUFA could overcome the inhibitory effects of PUFA was examined by using mixtures of FA pairs. Where a PUFA was used in combination with either stearic acid or oleic acid, there was some reduction in the inhibitory effects of the PUFA, but neither the saturated FA nor the MUFA was able to overcome the inhibition fully. This was the case even when the two FAs were used in equimolar ratios and the PUFA was used at a concentration of 15 $\mu$M, where the inhibitory effects were submaximal.

To investigate this phenomenon further, stearic acid was tested across a reciprocal range of concentrations against linoleic acid or EPA. Stearic acid was found to modulate the inhibitory effects of both PUFAs and reduced the growth inhibitory effects of both the *n*-3 and *n*-6 PUFAs to some extent, even when present as only 10% of the total FA (Figure 3). By contrast, the inhibitory effects of linoleic acid and EPA were found to be additive, such that the total concentration of PUFA determined the degree of inhibition of thymidine incorporation (Figure 3).

### *Effect of multiple FA combinations on cell proliferation*

Where the total FA concentration was limited to 30 $\mu$M, there was a significant decrease in thymidine incorporation in FA concentrations in which PUFA was the major component(s) ($\geqslant$70% of total). The effect was independent of whether the PUFA was predominantly in the form of *n*-3 or *n*-6 (Figure 4). At 100 $\mu$M total FA concentration, the inhibition of thymidine incorporation also became evident in the 'diet' mixture in which PUFA accounted for 50% of control. However, where the majority of the mixture was in the form of saturated or mono-unsaturated FA, there was no significant difference from the no FA control at either 30 or 100 $\mu$M total FA (Figure 4).

### Discussion

There is a consensus in the literature that PUFAs inhibit the growth of carcinoma cells *in vitro*. However, it is less clear whether these effects relate to cell proliferation rate *per se*, or to a consequence of the malignant transformation process of epithelial cells. Although several studies have shown

**Figure 3** Effects of reciprocal concentrations of fatty acids (bar charts), compared with the effects of the single fatty acids (symbols and dotted lines), on [$^3$H]thymidine incorporation. Results are expressed as growth indices relative to the BSA-only controls and means±95% confidence intervals are plotted.

**Figure 4** Effect of complex FA mixtures on urothelial cell proliferation. Mixtures of four FAs totalling final concentrations of either 30 $\mu$M (**a**) or 100 $\mu$M (**b**) were used representative of specific diets (see Table I). Results are expressed as growth indices relative to BSA-only controls and means±95% confidence intervals are plotted. $^*P<0.001$ by *t*-test. $^{**}P<0.001$ by *t*-test and ANOVA with Bonferroni correction.

differential effects of FAs on human transformed *vs* non-transformed cells *in vitro*, such studies have either related the effects of FA on carcinoma cells to fibroblasts (Begin *et al.*, 1986) or to an immortalised breast carcinoma-derived cell line (Grammatikos *et al.*, 1994) as the 'non-cancerous' counterpart. Clearly, neither case can offer a true reflection of the effects of FAs on normal epithelial cell proliferation. Our study is one of the first to report on the *in vitro* effects of FAs on normal human adult epithelial cells and our observations suggest that there are marked similarities in the responses to FAs of normal proliferating epithelial cells compared with studies involving carcinoma-derived cell lines. This is in agreement with studies of the effects of dietary FAs on proliferation of normal cells *in vivo*, which have also demonstrated that diets high in PUFAs can be anti-mitotic for the normally rapidly proliferating epithelial cells in intestinal crypts (Anti *et al.*, 1992; Bartram *et al.*, 1993; Pell *et al.*, 1994).

We have used free (non-esterified) FAs in the form of albumin-bound complexes as this is the physiological mode of presentation to normal cells *in vitro*. This hinders comparison with other approaches, such as the use of FAs in ethanol solution (Rose and Connolly, 1990; DeKoch *et al.*, 1994; Hatala *et al.*, 1994; Sylvester *et al.*, 1994; Maehle *et al.*, 1995). Nevertheless, almost all authors have reported that both *n*-3 and *n*-6 PUFAs inhibit growth of cells *in vitro* in an irreversible, dose-dependent manner. The one exception is the study of Sylvester *et al.* (1994), which showed stearic acid to be growth inhibitory and *n*-6 arachidonic acid to be stimulatory in mouse mammary epithelial cells respectively. In agreement with the observations made by Falconer *et al.* (1994) on human pancreatic adenocarcinoma cell lines, it appears that, at low concentrations, FAs may show a small enhancement of epithelial cell proliferation. Furthermore, in both this and the study by Falconer *et al.* (1994), different FAs were shown to interact to modulate overall cell response, implying that the actual composition of FAs in a diet may be significant. Specifically, we found the inhibitory effects of PUFAs to be additive, whereas addition of saturated FAs or MUFAs could, to some extent, overcome the inhibitory effects of PUFAs. This resembles the oleic acid-inducible reversal of the cytotoxic effects on pancreatic carcinoma cells reported by Falconer *et al.* (1994), although they found no effects with stearic acid.

From our data and the observations of others, we suggest that epithelial cell sensitivity to the cytostatic effects of PUFAs may be a function of proliferative activity and hence is not necessarily related to malignant transformation. Although the urothelium is a very slow turnover epithelium *in situ*, it is capable of very rapid proliferation rates both during regeneration *in vivo* (Marceau, 1990) and when grown *in vitro*, where we have shown the doubling time to be in the order of 15 h (Southgate *et al.*, 1994). Hence, in studies where normal, immortalised or cancerous cell lines have been compared (Cantril *et al.*, 1993; Grammatikos *et al.*, 1994), it was not clear whether there are underlying differences in proliferation rates which may account for differences in sensitivity to the cytostatic and cytotoxic effects of PUFAs.

It has been proposed that the differential effects of PUFAs on tumour cells may reflect decreased desaturase activity and hence an increased metabolic requirement for exogenous FAs (discussed by Grammatikos *et al.*, 1994). However, in normal peripheral blood lymphocytes, where dividing and non-dividing cell populations can be compared, it has been shown that mitogenesis is accompanied by a rapid and sustained increase in the polyunsaturation of the plasma membrane (Shires *et al.*, 1989). Hence, apparent differences in metabolism between normal and tumour cells may reflect proliferation rates, rather than any intrinsic property of carcinoma cells. Although our study was not designed to address the differential effects of *n*-3 and *n*-6 PUFAs on tumorigenesis, the data do suggest that *n*-6 PUFAs are more cytostatic to normal cells than *n*-3 PUFAs of an equivalent 18 carbon chain length. There was also a closer similarity in the dose-related response characteristics of normal urothelial cells to the $_{18}C$ mono-unsaturated oleic and $C_{18}$ *n*-3 ALA, than to either of the $C_{18}$ *n*-6 PUFAs tested.

As noted above, the *in vivo* data would suggest that diets high in fish oils might protect against tumour development (Anti *et al.*, 1992) and growth (Narisawa *et al.*, 1994) and can inhibit normal epithelial cell proliferation in colonic crypts (Bartram *et al.*, 1993; Pell *et al.*, 1994). Although the effects of fish oils have been attributed to the *n*-3 PUFAs, it must be remembered that fish oils typically contain less than 50% of *n*-3 PUFAs and it has been pointed out that care should be taken when attributing effects to single fatty acids when complex mixtures of oils are administered (Garton, 1992). In our experiments with complex mixtures, effects attributable to PUFAs were only observed where the PUFAs contributed to more than 50% of the total fatty acids and were very much more pronounced at high FA concentrations. Our data suggest that PUFAs of both *n*-3 and *n*-6 types may be growth inhibitory and therefore any differential effects of *n*-3 *vs* *n*-6 PUFAs on cell growth *in vivo* might be attributable to additional factors.

In addition to a potential role in colon cancer prevention (Anti *et al.*, 1992), there is considerable interest in exploiting the dietary effects of *n*-3 PUFAs as an adjunct to conventional cancer therapy (reviewed by Das, 1990; Burns and Spector, 1994). However, our findings advocate caution if the targets are proliferating rather than specific tumour cell populations, as such an approach would offer no advantage over other conventional chemotherapy or radiotherapy regimens. In this context, it is of interest that the PUFAs appeared to irreversibly arrest growth in the normal cells in a way that is very reminiscent of mitomycin-C treatment or radiation-induced 'reproductive cell death', resulting in intact monolayers of non-dividing cells. Although the specific cytostatic mechanism was not explored here, it has been suggested that increased free radical generation through lipid peroxidation may be involved (Begin *et al.*, 1986; 1988). It has been reported that short-chain FAs may kill colonic tumour cells by inducing apoptosis (Hague *et al.*, 1995). Using morphological criteria, we did not observe apoptotic cells in normal urothelial cells treated with high concentrations of long-chain (18–22 carbon) PUFAs. Although further specific studies would be required, these observations suggest that short-chain FAs may have a different mode of action or induce apoptosis specifically in malignantly transformed cells. Alternatively, this may reflect a biological difference between epithelial cells of intestinal and urothelial origins, as there is good evidence that apoptosis is a central mechanism for normal epithelial cell turnover in the intestinal tract (Hall *et al.*, 1994).

In conclusion, we suggest that normal human epithelial cells show considerable sensitivity to PUFAs that probably relates to proliferation rate *in vitro*, rather than reflecting any intrinsic differences between epithelial cells and their malignantly transformed carcinoma counterparts.

**Acknowledgements**
We thank Wendy Kennedy and Sarah Derrett for technical assistance. The work was supported by a contract from the Ministry of Agriculture, Fisheries and Food. We also acknowledge the support of the Imperial Cancer Research Fund.

### References

AGREN JJ, TORMALA M-L, NENONEN MT AND HANNINEN O. (1995). Fatty acid composition of erythrocyte, platelet and serum lipids in strict vegans. *Lipids*, **30**, 365–369.

ANTI M, MARRA G, ARMELAO F, BARTOLI GM, FICARELLI R, PERCESEPE A, DE VITIS I, MARIA G, SOFO L, RAPACCINI GL, GENTOLINI N, PICCIONI E AND MIGGIANO G. (1992). Effect of omega-3 fatty acids on rectal mucosal cell proliferation in subjects at risk for colon cancer. *Gastroenterology*, **103**, 883–891.

BARTRAM H-P, GOSTNER A, SCHEPPACH W, REDDY BS, RAO CV, DUSEL G, RICHTER F, RICHTER A AND KASPER H. (1993). Effects of fish oil in rectal cell proliferation, fatty acids and prostaglandin $E_2$ release in healthy subjects. *Gastroenterology*, **105**, 1317–1322.

BEGIN ME, ELLS G, DAS UN AND HORROBIN DF. (1986). Differential killing of human carcinoma cells supplemented with n-3 and n-6 polyunsaturated fatty acids. *J. Natl Cancer Inst.*, **77**, 1053–1062.

BEGIN ME, ELLS G AND HORROBIN DF. (1988). Polyunsaturated fatty acid-induced cytotoxicity against tumor cells and its relationship to lipid peroxidation. *J. Natl Cancer Inst.*, **80**, 188–194.

BURNS CP AND SPECTOR AA. (1994). Biochemical effects of lipids on cancer therapy. *J. Nutr. Biochem.*, **5**, 114–123.

CANTRILL RC, ELLS GW AND HORROBIN DF. (1993). Mechanisms of action of cytotoxic fatty acids. *S. Afr. J. Science*, **89**, 398–400.

DAS UN. (1990). Gamma-linolenic acid, arachidonic acid, and eicosapentaenoic acid as potential anticancer drugs. *Nutrition*, **6**, 429–434.

DE KOCK M, LOTTERING M AND SEEGERS JC. (1994). Differential cytotoxic effects of gamma-linolenic acid on MG-63 and HeLa cells. *Prostaglandins Leukot. Essent. Fatty Acids*, **51**, 109–120.

DOLECK T AND GRANDITIS G. (1991). Dietary polyunsaturated fatty acids and mortality in the multiple risk factor intervention trial (MRFIT). *World Rev. Nutr. Diet.*, **66**, 205–216.

DOUGHERTY RM, GALLI C, FERRO-LUZZI A AND IACONO JM. (1987). Lipid and phospholipid fatty acid composition of plasma, red blood cells and platelets and how they are affected by dietary lipids: a study of normal subjects from Italy, Finland and the USA. *Am. J. Clin. Nutr.*, **45**, 443–455.

FALCONER JS, ROSS JA, FEARON KCH, HAWKINS RA, O'RIORDIAN MG AND CARTER DC. (1994). Effect of eicosapentaenoic acid and other fatty acids on the growth *in vitro* of human pancreatic cancer cell lines. *Br. J. Cancer*, **69**, 826–832.

GARTON A. (1992). *Unsaturated Fatty Acids: Nutritional and Physiological Significance. The Report of the British Nutrition Foundation's Task Force*. Chapman & Hall: London.

GRAMMATIKOS SI, SUBBAIAH PV, VICTOR TA AND MILLER WM. (1994). n-3 and n-6 fatty acid processing and growth effects in neoplastic and non-cancerous human mammary epithelial cell lines. *Br. J. Cancer*, **70**, 219–227.

GREGORY CD, DIVE C, HENDERSON S, SMITH CA, WILLIAMS GT, GORDON J AND RICKINSON AB. (1991). Activation of Epstein–Barr virus latent genes protects human B cells from death by apoptosis. *Nature*, **349**, 612–614.

HAGUE A, ELDER DGJ, HICKS DJ AND PARASEKEVA C. (1995). Apoptosis in colorectal tumour cells: induction by the short chain fatty acids butyrate, propionate and acetate and by the bile salt deoxycholate. *Int. J. Cancer*, **60**, 400–406.

HALL PA, COATES PJ, ANSARI B AND HOPWOOD D. (1994). Regulation of cell number in the mammalian gastrointestinal tract: the importance of apoptosis. *J. Cell Sci.*, **107**, 3569–3577.

HATALA MA, RAYBURN J AND ROSE DP. (1994). Comparison of linoleic acid and eicosapentaenoic acid incorporation into human breast cancer cells. *Lipids*, **29**, 831–837.

HUTTON KAR, TREJDOSIEWICZ LK, THOMAS DFM AND SOUTHGATE J. (1993). Urothelial tissue culture for bladder reconstruction: an experimental study. *J. Urol.*, **150**, 721–725.

KROMHOUT D. (1990). The importance of n-6 and n-3 fatty acids in carcinogenesis. *Med. Oncol. Tumor. Pharmacother.*, **7**, 173–176.

LENTNER C. (1984). *Geigy Scientific Tables*, **3**, Giba Geigy Publishers: Basle, Switzerland.

MAEHLE L, EILERTSEN E, MOLLERUP S, SCHONBERG S, KROKAN HE AND HAUGEN A. (1995). Effects of n-3 fatty acids during neoplastic progression and comparison of *in vitro* and *in vivo* sensitivity of two human tumour cell lines. *Br. J. Cancer*, **71**, 691–696.

MARCEAU N. (1990). Cell lineages and differentiation programs in epidermal, urothelial and hepatic tissues and their neoplasms. *Lab. Invest.*, **63**, 4–20.

NARISAWA I, TAKAHASHI M, KOTANAGI H, KUSAKA H, YAMAZAKI Y, KOYAMA H, FUKAURA Y, NISHIZAWA Y, KOTSUGAI M, ISODA Y, HIRANO J AND TANIDA N. (1991). Inhibitory effect of dietary perilla oil rich in the n-3 polyunsaturated fatty acid alpha-linolenic acid on colon carcinogenesis in rats. *Jpn. J. Cancer Res.*, **82**, 1089–1096.

NARISAWA T, FUKAURA Y, YAZAWA K, ISHIKAWA C, ISODA Y AND NISHIZAWA Y. (1994). Colon cancer prevention with a small amount of dietary perilla oil high in alpha-linolenic acid in an animal model. *Cancer*, **73**, 2069–2075.

PARKIN DM, PISANI P AND FERLAY J. (1993). Estimates of the worldwide incidence of eighteen major cancers in 1985. *Int. J. Cancer*, **54**, 594–606.

PELL JD, BROWN JC AND JOHNSON IT. (1994). Polyunsaturated fatty acids of the n-3 series influence intestinal crypt cell production in rats. *Carcinogenesis*, **15**, 1115–1119.

PRESTON-MARTIN S, PIKE MC, ROSS RK, JONES PA AND HENDERSON BE. (1990). Increased cell division as a cause of human cancer. *Cancer Res.*, **50**, 7415–7421.

REDDY BS. (1994). Chemoprevention of colon cancer by dietary fatty acids. *Cancer Metast. Rev.*, **13**, 285–302.

ROSE DP AND CONNOLLY JM. (1990). Effects of fatty acids and inhibitors of eicosanoid synthesis on the growth of a human breast cancer cell line in culture. *Cancer Res.*, **50**, 7139–7144.

SHIRES SE, KELLEHER J AND TREJDOSIEWICZ LK. (1989). Effects of linoleic acid and mitogenic stimulation on the fatty acid composition of human lymphocytes. *Biochim. Biophys. Acta*, **1002**, 74–78.

SOUTHGATE J, HUTTON KAR, THOMAS DFM AND TREJDOSIEWICZ LK. (1994). Normal human urothelial cells *in vitro*: proliferation and induction of stratification. *Lab. Invest.*, **71**, 583–594.

SPECTOR AA, JOHN K AND FLETCHER JE. (1969). Binding of long chain fatty acids to bovine serum albumin. *J. Lipid Res.*, **10**, 56–67.

SYLVESTER PW, BIRKENFELD HP, HOSICK HL AND BRISKI KP. (1994). Fatty acid modulation of epidermal growth factor-induced mouse mammary epithelial cell proliferation *in vitro*. *Exp. Cell Res.*, **214**, 145–153.

WHELAN SL, PARKIN DM AND MASUYER E. (1990). *Patterns of Cancer in Five Continents*. International Agency for Research on Cancer: Lyon.